# Brain Tumor Infodemiology: Worldwide Online Health-Seeking Behavior Using Google Trends and Wikipedia Pageviews

**DOI:** 10.3389/fonc.2022.855534

**Published:** 2022-04-19

**Authors:** Mark Willy L. Mondia, Adrian I. Espiritu, Roland Dominic G. Jamora

**Affiliations:** ^1^ Department of Neurosciences, College of Medicine and Philippine General Hospital, University of the Philippines Manila, Manila, Philippines; ^2^ Department of Clinical Epidemiology, College of Medicine, University of the Philippines Manila, Manila, Philippines; ^3^ Institute for Neurosciences, St. Luke’s Medical Center, Quezon City & Global City, Philippines

**Keywords:** google trends, wikipedia, brain tumor, glioblastoma, infodemiology

## Abstract

**Background:**

Searching the internet for information on common neurologic symptoms and diseases has been increasing in recent times. It is postulated that online search volume data could be utilized to gauge public awareness and real-world epidemiological data regarding brain tumors.

**Objectives:**

The goal of this study was to describe the pattern of online search queries of keywords related to neoplasms of the central nervous system (CNS).

**Methods:**

Using Google Trends, search activity from January 2004 – January 2021 was quantified using relative search volume (RSV). The average RSV for the first 3 years was compared with the final 3 years to account for percent change. Wikipedia article views from July 2007 – January 2021 were generated using Pageviews. Peaks in RSV and page views were then matched for related news.

**Results:**

“Brain tumor”, “brain cancer”, “glioblastoma”, and “glioma” had the highest search volume. RSV from Google Trends and views of Wikipedia pages reflected comparable data in terms of known prevalence rankings of tumor subtypes. There were no observable trends that could correlate to the rising numbers of brain tumor cases worldwide. However, headlines of personalities being diagnosed with glioblastomas were mostly responsible for temporary increases in public interest.

**Conclusions:**

Transient rises in online search volume mirror public awareness of more aggressive CNS neoplasms that have a high burden of disease. Worldwide interest in brain tumors may not necessarily correspond to clinical occurrence, but may signify an unmet gap in providing accurate online information to neuro-oncologic patients.

## Introduction

Trends in health informatics that deal with internet usage *via* search terms called “big data”, are increasingly being used to approximate real-time statistics on disease epidemiology, healthcare research, public knowledge, and patient health-seeking behavior (1, 2). Big data analytics in healthcare and medicine refers to processing information from thousands of patient records to extrapolate possible correlations as well as developing predictive models with the use of data mining techniques (3). On a larger scale, “big data” encompasses available information published in an open access format like the internet – which could sum up to millions of data points. This resulted in a growing new research discipline called infodemiology (4). This term is a combination of information and epidemiology and was defined by Eysenbach as “the science of distribution and determinants of information in an electronic medium, specifically the internet, or in a population, with the ultimate aim to inform public health and public policy” (5).

According to a 2019 review article, Google Trends is one of the highly utilized tools in addressing health issues and topics using data extracted from the internet (2). Google Trends is a free and public online feature of Google Inc., which analyzes users’ search queries and generates geospatial and temporal patterns in search volumes for user-specific terms (1, 2). Certain flaws on over- and under-estimation have been cited for Google Trends, thus necessitating complementary big data from free online encyclopedia services like Wikipedia (4, 6).

There is a beginning paradigm shift in the impact of the internet in shaping general public awareness and healthcare delivery in the neurological setting (7). Infodemiological studies on epilepsy (8–12), stroke (13), multiple sclerosis (14, 15), poliomyelitis (16), meningitis (17), Alzheimer disease (18), movement disorders (19), and even on telerehabilitation (20) and teleneurology (21) have shown an unsubstantial connection between actual incidence and prevalence, but presented initial trends that reflect the increasing number of people using the internet to seek online health information regarding these neurological diseases.

The global incidence of brain and spinal tumors, though relatively less common than other neurological diseases, varied by region and economic status according to the latest 2019 data – with lower incidences in Eastern and middle to low-income countries (22). However, based on the 2020 statistics on worldwide internet usage, Asia and Africa have the biggest shares with 55.1% and 17.2%, respectively, wherein East Asia having the most number of internet users at 1.1 billion (23, 24). Additionally, “cancer” is one of the top three health-related internet searches and is the most common source of information for patients about their disease (25, 26). Taken together, big data analysis is theoretically useful in assessing brain tumor statistics, where gaps in data gathering partly due to its relatively low epidemiology could be addressed by assessing online figures.

Our study, therefore, evaluated and interpreted internet search queries for terms related to brain tumors using Google Trends and Wikipedia article views.

## Methods

### Google Search

#### Search Strategy

The following search terms were entered in Google Trends main page (see http://www.google.com/trends): “Brain tumor”, “Brain cancer”, “Central nervous system (CNS) tumor”, “Glioma”, “Glioblastoma”, “Astrocytoma”, “Oligodendroglioma”, “CNS Lymphoma”, “Medulloblastoma”, and “Meningioma”. These terms were chosen to cover for the most common histopathologic diagnosis of both benign and malignant primary brain tumors (27, 28). “Temozolomide” was the only drug included since it’s the most specific chemotherapy used for primarily for brain tumors (29). All searches were conducted on January 20, 2021.

#### General Search Prevalence

The aforementioned search term keywords were also entered into the main search engine (Google main page, see http://www.google.com) to obtain the absolute number of occurrences in the entire database over time. This data reflects the information prevalence, which is an infodemiological indicator of the occurrence of a keyword or concept in an electronic medium, particularly the internet (5).

#### Google Trends Analysis

Google Trends gives access to real-time data from January 2004 up to 36 hours before the search is conducted (2). The search settings were as follows: “Worldwide”, “since 2004”, and “All categories”. Data obtained from Google Trends is reported as relative search volume (RSV) on a scale from 0 to 100, wherein the values are normalized over the selected time frame (2). Thus, 0 would indicate very low search volumes (but not necessarily no searches) and 100 would represent the highest relative use of the term over time (2, 30). This ensures that search results are comparable and proportionate to the time and location of the query (2, 19). If available, the “Topic”, “Disease”, “Medical Condition”, or “Medication” options were included in the analysis. These options take into consideration related terms to the search query that share the same concept for any language. Percentage growth of the search terms was computed by comparing the first 3-year epoch values of search terms to the latest 3-year epoch giving (19).

### Wikipedia Search

#### Data Collection and Wikipedia Trend Analysis

Information on Wikipedia page visits using the same search term were obtained using the Pageviews option as part of the Wikimedia Statistics data page (30). This tool reports trends in the number of article views per day regarding specific Wikipedia search terms starting from July 1, 2015 (12, 19). All searches were conducted on January 23, 2021. Any peaks in views were counterchecked with any news related to the keyword *via* Google search to determine if there was a relationship with the surge in page views and publication of certain headlines (19).

### Statistical Analysis

Data from both Google Trends and Wikipedia Pageviews were tabulated and graphically represented. Linear regression was used to determine trend, while presence or absence of correlation was determined use the Pearson coefficient (*R)* and deemed significant for *p*-value <0.05.

## Results

### Google Search Trends

**Table 1** demonstrates the average RSV and information prevalence for the keywords searched. The terms with the highest information prevalence were “brain cancer”, “brain tumor”, and “nervous system tumor” with the lowest to “oligodendroglioma”, “temozolomide”, and “medulloblastoma”. Average RSV takes into account monthly RSVs since January 2004, where “brain tumor” (disease), “meningioma” (medical condition), and “glioblastoma” (genetic disorder) recorded the highest values at 76, 65, and 42, respectively. “Glioblastoma multiforme”, “CNS lymphoma”, and “nervous system tumor” had the lowest average RSVs at 5, 5, and <1, respectively.

**Table 1:** Average relative search volume (RSV) and information prevalence from Google search and trends.

Google search term	Information prevalence	Overall average RSV	First 3 years epoch average RSV	Final 3 years epoch average RSV	Percent change in RSV
“Brain cancer”	789,000,000	14	12	15	+25
“Brain tumor”	144,000,000	23	20	25	+25.0
“Nervous System Tumor”	81,000,000	<1	<1	<1	0
“CNS Lymphoma”	17,4000,000	5	5	12	+58.3
“Glioma”	10,900,000	14	17	10	-41.2
“Glioblastoma”	7,760,000	22	24	21	-12.5
“Glioblastoma Multiforme”	5,870,000	5	7	3	-57.1
“Meningioma”	3,490,000	40	31	24	-22.6
“Astrocytoma”	3,310,000	26	29	47	+38.3
“Medulloblastoma”	1,810,000	7	7	4	-42.9
“Temozolomide”	1,390,000	10	8	26	+69.2
“Oligodendroglioma”	814,000	9	9	16	+43.8
“Brain tumor” (Disease)	N/A	76	65	77	+18.5
“Meningioma” (Medical Condition)	N/A	65	54	39	-27.8
“Glioblastoma” (Genetic Disorder)	N/A	42	47	34	-27.7
“Astrocytoma” (Topic)	N/A	40	43	66	+34.8
“Temozolomide” (Medication)	N/A	35	26	67	+61.2
“Glioma” (Topic)	N/A	22	25	16	-36.0
“Oligodendroglioma” (Topic)	N/A	11	11	21	+47.6
“Medulloblastoma” (Topic)	N/A	12	12	6	-50.0

N/A, Not applicable.

In terms of comparison of the first 3 and last 3 years average RSVs, search volume reduction was seen in the following terms: “glioma”, “glioma” (topic), “glioblastoma”, “glioblastoma” (genetic disorder), “glioblastoma multiforme”, “medulloblastoma”, “meningioma”, and “meningioma” (medical condition). RSV increase was noted in the following terms: “brain tumor”, “brain tumor (disease), “brain cancer”, “astrocytoma”, “astrocytoma” (topic), “oligodendroglioma”, “oligodendroglioma” (topic), and “CNS lymphoma”. “Temozolomide” had the highest percent increase at 69.2%, while “medulloblastoma” (topic) had the greatest percent decrease at 50%.

**Figure 1:**
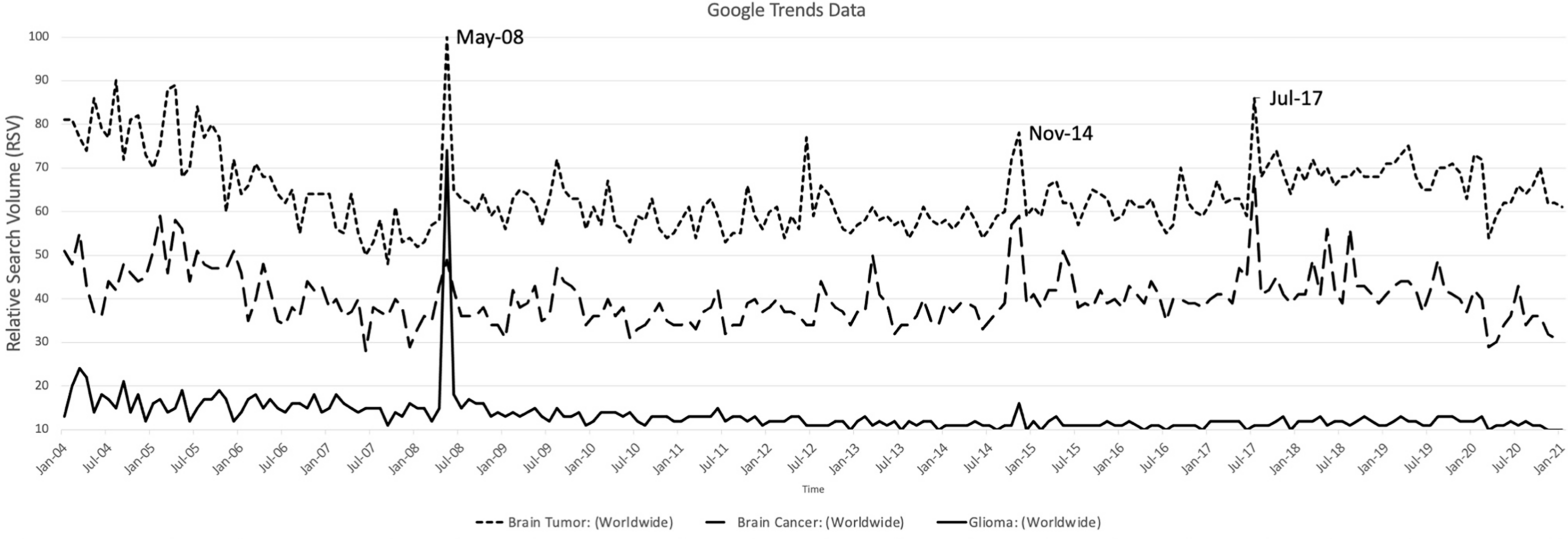
Relative search volumes (RSV) obtained from Google Trends for “Brain tumor”, “Brain Cancer” and “Glioma” plotted against time from January 2004 to January 2020. Labelled are the significant peaks in RSV shared by the presented search terms

Monthly RSVs of “brain tumor”, “brain cancer”, “glioma”, “glioblastoma”, and “meningioma” were graphed against time as shown in **Figure 1**. There were three standout peaks in RSVs during May 2008 (“brain tumor”: 100; “glioma”:76; “brain cancer”: 51), November 2014 (“brain tumor”: 82, “brain cancer”: 64), and July 2017 (“brain tumor”: 89, “brain cancer”: 72).

Interest per region and top related searches were also tabulated in **Supplementary Table 1**. Most of the keywords analyzed were searched from the United States, Australia, and Canada. Top related queries were mostly permutations of the keywords used as well as symptoms and treatment for the specific types of brain tumors.

### Wikipedia Search Trends

**Figure 2:**
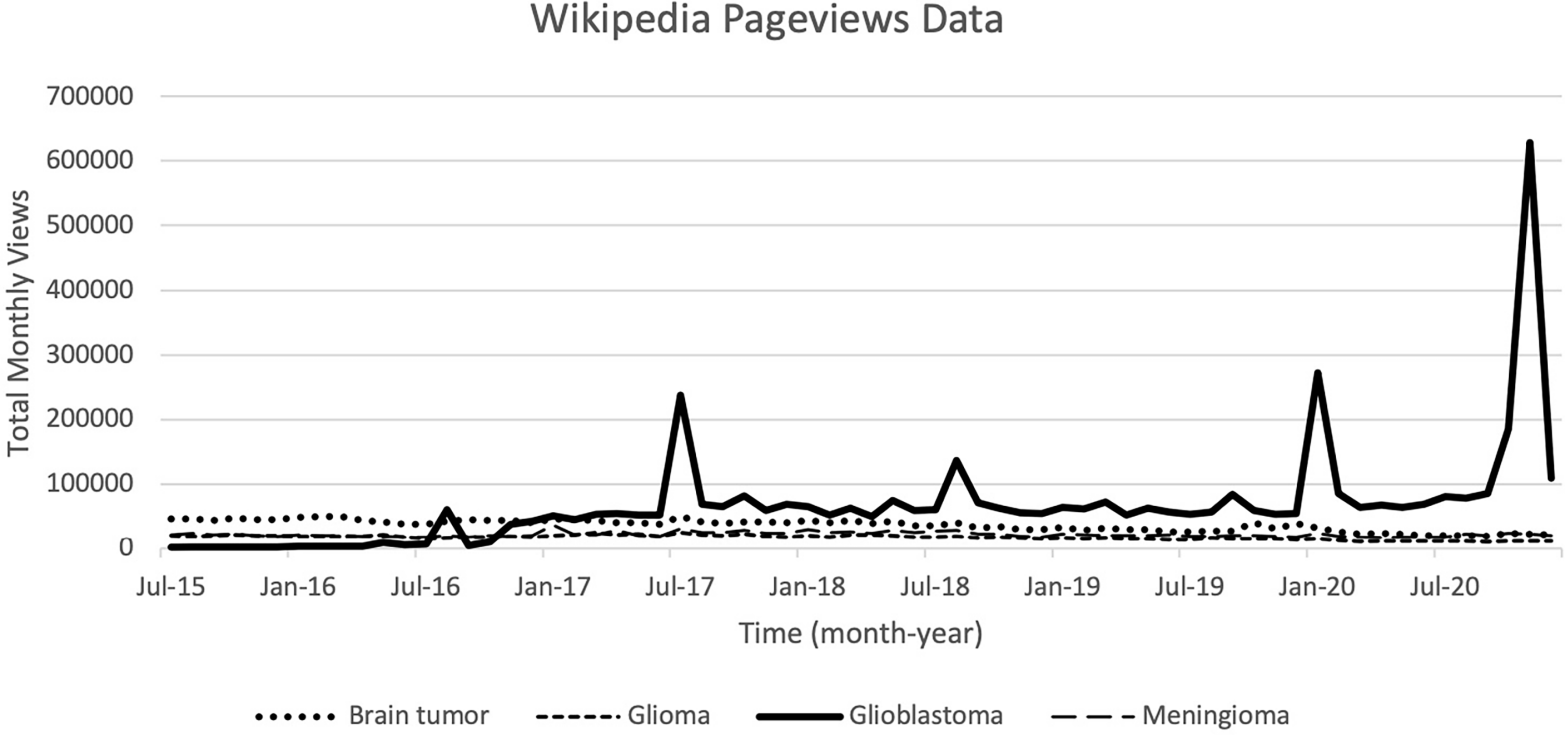
Trends of Wikipedia Pageviews for selected search term keywords plotted against time since July 2015.

As seen in **Table 2**, top pageviews in Wikipedia from keywords searched were “glioblastoma”, “brain tumor”, and “meningioma”, while “brain cancer”, “CNS lymphoma” and “glioblastoma multiforme” had the lowest page views. The pages that had more edits done were also viewed the most times. Trends in Pageviews were presented in **Figure 2** wherein only the search term for “glioblastoma” had appreciable peaks during August 2016, July 2017, August 2018, and January 2020, with the highest peak last November 2020. The rest of the keywords showed no peaks in views.

**Table 2:** Tabulated Pageviews Data from Wikipedia searches.

Page Title	Total views	Monthly average views	Edits	Editors
Glioblastoma	4,419,751	66,966	556	210
Brain tumor	2,391,649	36,237	584	286
Meningioma	1,436,828	21,770	157	66
Glioma	1,140,765	17,284	173	87
Astrocytoma	837,149	12,684	74	51
Medulloblastoma	612,350	9,278	84	60
Temozolomide	585,193	8,867	80	51
Oligodendroglioma	414,937	6,287	65	35
Brain cancer	329,970	5,000	12	9
CNS lymphoma	8,849	134	0	0
Glioblastoma multiforme	8,292	126	1	1

Peaks in Google Trends and Wikipedia Pageviews were tabulated with corresponding news articles during that particular month related to the keyword (see **Table 3**). “Brain tumor”, “brain cancer”, “glioblastoma”, and “glioma” were the terms consistently peaking in Google Trends, while only “glioblastoma” had peaks in Wikipedia Pageviews. Once the maximal values in views and searches were achieved, values would abruptly return to baseline – a pattern common to isolated incidences rather than slowly increasing trends that are reflective of recent brain tumor burden of disease. Consistent throughout the headlines were articles regarding diagnosis and death of mostly American political (i.e. Ted Kennedy, John McCain, and Beau Biden) and entertainment/sports personalities (i.e. reporters, singers, or athletes). There were more articles related to innovative treatment and research on brain tumors from 2018-2020.

**Table 3:** Relation of peaks in Google Trends and Wikipedia Pageviews with online news headlines.

Search terms	Month and peak year	Peak-related online headline (date of publication)
Brain tumor (100 RSV) Brain cancer (51 RSV) Glioma (76 RSV)	May 2008	• Senator Edward Kennedy Has Malignant Brain tumor (May 20, 2008)
Brain tumor (82 RSV) Brain cancer (37 RSV) Glioblastoma (19 RSV)	June 2012	• Sheryl Crow’s Tumor Unlikely Cause of Memory Loss, Experts Say (June 6, 2012) • Arizona Murder-Suicide Dad A Brain Tumor (June 8, 2012)
Brain tumor (6 RSV) Brain cancer (57 RSV) Glioblastoma (19 RSV)	March 2013	• Occupational radiation exposure linked to left-sided brain tumors (March 4, 2013) • Valerie Harper’s Rare Brain Cancer: What Caused It? (March 6, 2013) • Dave Weaver: Skateboarding, Buffalo & Battling Brain Cancer (March 29, 2013)
Brain tumor (82 RSV) Brain cancer (64 RSV) Glioblastoma (45 RSV)	November 2014	• Brittany Maynard’s Death: Why Is Brain Cancer So Lethal? (November 5, 2014) • Former Queensland premier Wayne Gross dies (November 11, 2014) • Cannabis extracts can “dramatically slow” growth of brain cancer tumours, new research suggests (November 15, 2014) • Electrical Scalp Device Can Slow Progression of Deadly Brain Tumors (November 15, 2014)
Brain tumor (63 RSV) Brain cancer (55 RSV) Glioblastoma (22 RSV)	May 2015	• Beau Biden, son of vice president, dies of brain cancer (May 30, 2015)
Glioblastoma (60,060 Pageviews; 24 RSV)	August 2016	• After Overcoming a Brain Tumor, Andrew Leahey Returns to St. Louis with a New Outlook on Music (August 1,2016) • Do mobile phones give you brain cancer? (August 8, 2016) • Journalist With Brain Cancer Who Penned Her Final Column Last Month Dies (August 14, 2016) • Edmonds woman battling brain cancer to be featured in TV documentary this week (August 16, 2016) • To help cancer patients, lawmakers pushed access to a controversial doctor (August 29, 2016) • The laser probe, the iKnife, and the cutting edge of surgery (August 31, 2016)
Brain tumor (89 RSV) Brain cancer (72 RSV) Glioblastoma (237,209 Pageviews; 64 RSV) Glioma (12 RSV)	July 2017	• Maria Menounos recovering from brain tumor surgery (July 4, 2017) • Sen. John McCain has brain cancer, aggressive tumor surgically removed (July 20, 2017) • Meet the Fort Lee woman who survived glioblastoma (July 20, 2017) • These experimental treatments target brain cancer like John McCain’s (July 26, 2017)
Glioblastoma (136,951 Pageviews; 45 RSV) Brain cancer (57 RSV)	August 2018	• She made a career out of studying the brain. Then hers veered off course (August 6, 2018) • After One Writer Finds Out She Has a Brain Tumor, She Confronts It Head On (August 10, 2018) • MIT Creates AI to Optimize Brain Cancer Treatment (August 13, 2018) • Brain cancer dataset now available free to researches worldwide (August 20, 2018) • Lasers help fight deadly brain tumors (August 22, 2018) • John McCain: The Incredible Life of an American Hero (August 27, 2018) • Brain tumour not stopping boy from living life to the fullest (August 28, 2021) • How tiny metal beads could make chemotherapy more effective for brain tumours (August 31, 2018)
Glioblastoma (272,584 Pageviews)	January 2020	• Mount Sinai receives more than $10 million in grant funding for brain tumor research (January 2, 2020) • A.I. Comes to the Operating Room (January 6, 2020) • Brain tumor research could help future precision medicine (January 7, 2020) • AI matches humans at diagnosing brain cancer from tumor biopsy images (January 8, 2020) • Niel Peart, Rush drummer and lyricist, has died at age 67 (January 10, 2020) • YouTube vlogger Samantha Jaelle dies of brain tumor (January 13, 2020) • Blackstone Valley Tech football player Tony Pena dies from brain cancer (January 15, 2020) • Mobile phones cause tumours, Italian court rules, in defiance of evidence (January 15, 2020) • Improved Microfluidic Brain Cancer Chip Tests and Analyzes Chemotherapies (January 17, 2020) • Living with Brain Cancer: Embracing a Difficult Diagnosis (January 20, 2020) • Taylor Swift reveals her mother has been diagnosed with a brain tumor (January 21, 2020) • Stem cells, CRISPR and gene sequencing technology are basis of new brain cancer model (January 28, 2020) • Pro Football Hall of Famer Chris Doleman dies at 58 following battle with brain cancer (January 29, 2020) Glioblastoma Alters Synaptic Function to Promote Tumor Growth (January 30, 2020)
Glioblastoma (628,268 Pageviews)	November 2020	• Joe Biden visits son Beau’s grave on Election Day morning (November 4, 2020) • Inside Taylor Swift’s Mom’s Cancer Battle (November 5. 2020) • Gammatiles Zap Brain Cancer (November 7, 2020) • With Growing Incidence of Brain Cancer, The Brain Tumor Drug Market Could Reach $3.4 Billion in 2022 (November 17, 2020) • Eddie Van Halen Had Stage 4 Lung Cancer and a Brain Tumor, Reveals His Son Wolfgang (November 17, 2020) • Graduate, 22, with advanced brain cancer forced to fund raise £100,000 for German treatment (November 17, 2020) • Jason Mamoa calls Marshfield boy (and Aquaman superfan) fighting cancer (November 18, 2020) • CNS Pharmaceuticals Announces FDA Filing for Investigational New Drug To Treat Glioblastoma (November 19, 2020) • Medicenna Announces Upcoming Oral Presentations at the 2020 Society for Neuro-Oncology Annual Meeting (November 18, 2020) • Russell Watson: Did I’m a Celebrity star have a brain tumour? (November 20, 2020) • Tempe brain tumor therapy company garners $16M in capital raise (November 23, 2020) • Brain cancer by Johnny Ruffo, Home and Way actor and X Factor star, sparks outpouring of celebrity support (November 24, 2020) • “So many kids are suffering”: Edmonton family donates son’s brain tumour to research (November 27, 2020) • Quincey grateful 1-year-old son recovering from surgery for brain tumor (November 25, 2020) • MindMatters: “I am One in 468”, American Brain Tumor Association Launches Public Awareness & Fundraising Campaign (November 2020)

RSV, relative search volume.

### Ancillary Search Terms

As seen in **Table 4**, spikes in search interest were seen for “Tumor treating fields” (TTF) on July 2017 and May 2019. When cross-referenced, these corresponded to the publication of Stupp et al. on the first randomized trial using TTF with temozolomide on recurrent GBMs as well as updates on TTF with gliomas and lung cancer during the 2019 American Society of Clinical Oncology meeting (29). A search for a more common term like the “brain” was done yielding an average RSV of 61 with range of 51-73 that fluctuates throughout the years, notably higher than the average RSV of more specific terms for brain tumors. There was only one outlier peak on January 2020, which was similar to data gathered for “brain tumors”. As seen in **Table 3**, this could be hypothesized to be related to entertainment-related news of brain cancer diagnosis.

**Table 4:** Infodemiological data on using “Tumor Treating Field” as a search term.

Date	Google Trends (RSV)
**2014-09**	59
**2015-07**	53
**2016-06**	49
**2016-09**	53
**2017-02**	100
**2017-07**	50
**2018-11**	44
**2019-07**	42
**2019-05**	84
**2019-06**	44
**2019-08**	93
**2019-09**	41
**2020-07**	38
**2020-09**	37
Date	Wikipedia Pageviews (Total Views: 396)
**2018-09**	7
**2018-10**	27
**2018-11**	18
**2018-12**	20
**2019-01**	22
**2019-02**	16
**2019-03**	18
**2019-04**	13
**2019-05**	12
**2019-06**	10
**2019-07**	16
**2019-08**	12
**2019-09**	21
**2019-10**	29
**2019-11**	13
**2019-12**	15
**2020-01**	8
**2020-02**	9
**2020-03**	10
**2020-04**	17
**2020-05**	11
**2020-06**	5
**2020-07**	11
**2020-08**	10
**2020-09**	11
**2020-10**	8
**2020-11**	10
**2020-12**	7
**2021-01**	10

RSV, relative search volume.

## Discussion

Seminal studies looking into online search behavior during influenza epidemics that correlated with peaks in the actual incidence of flu cases were pivotal to the establishment of infodemiology (31). Brigo, et al. applied this concept of health informatics to the field of neurology. They noted possible increasing interest in the use of Google and Wikipedia for definitions, symptomatology, natural history, and treatment of common neurological diseases (3, 7–21).

Our study applied a similar working framework to determine if infodemiological data regarding CNS neoplasms would show patterns that could be comparable to disease epidemiology and could be used as a surrogate to characterize the health-seeking behavior of patients. This was the first attempt to evaluate internet searches into common brain tumors and chemotherapeutic drugs. Our study identified a relatively stable search volume with identifiable peaks without showing significant negative or positive trends.

Data from Google Trends showed no significant differences in trends of RSVs for search terms involving specific types of brain tumors. Strictly speaking, there was no clear correspondence to the actual prevalence of tumor subtypes. The highest average RSVs belonged to “brain tumor”, “meningioma”, and “glioblastoma”. It should be noted though that meningioma is the most common benign solid intracranial tumor and glioblastoma is the most common malignant CNS neoplasm (27). However, related keywords like “glioblastoma multiforme” (same as glioblastomas) and “central nervous system tumors” (similar to brain tumor) had the lowest average RSVs, which could reflect the bias favoring less technical terms.

In terms of direct analysis of search trends to actual incidence, most large databases agree on a fluctuating trend in overall incidence of primary tumors of the brain and spine. A systematic review of epidemiological data from 1985 to 2014, which included 53 studies reported an overall incidence rate of all brain tumors to be 10.82 (95% CI: 8.63 – 13.56) per 100 000 person-years (32). The 2016 global burden of disease study saw a 17.3% increase from 1990 in age-standardized incidence rates of CNS cancer (21). On the other hand, the 2011-2015 Central Brain Tumor Registry of the United States (CBTRUS) statistical report has seen statistically significant increase in trends, but indicates that this might not necessarily represent a large change in proportion of individuals over time (33). Many factors influence these trends in the same manner as trends in internet search queries. The most identifiable difference is how actual incidence rates are more proportional to increases in overall world population over time versus only spikes in search hits seen in infodemiological data for CNS tumors.

Comparing initial and final 3-year epochs for trend determination was used in one study of epilepsy infodemiology and was adapted in this study as well (12). Most of the common tumor subtypes showed a decrease (glioma, meningioma), while less common subtypes (astrocytoma, oligodendroglioma, CNS lymphoma) had a general increase. One possible explanation is that histologic and molecular diagnoses are improving with time resulting to growing awareness for these terms, but in the process decreasing the share of more common tumor subtypes in a normalized scale like RSVs.

Google Trends also takes into account interest per region and as expected, western countries with big populations were almost always part of the top 10 regions that search about brain tumors. It is important to note that China, the top region in terms of internet users (24), being the most populated country and part of East Asia has not been accounted for. This is probably due to the sociopolitical stand of the government in online behavior as well as language differences. Arguably, this should have been addressed by the “topic” option of Google Trends that takes into consideration different languages. Other Asian countries that appear more than five times in the interest per region are South Korea (in 9/20 search terms), Philippines (in 6/20 search terms), and Singapore (in 5/20 search terms). Access to internet and cultural online habits could be reasons for higher search volume coming from these areas. Research outputs for brain tumors can be explored as possible sources of online search volumes for countries that dedicate more of their gross domestic product (GDP) to research and development like Singapore (34).

Wikipedia article views are expressed in absolute values and also show a trend that mimics the epidemiological data of brain tumor subtypes, with the exception of glioblastoma overtaking terms like brain tumor and meningioma. This could be explained by higher public awareness of glioblastomas due to their dismal prognosis and association with known personalities with such diagnosis. This was supported by the fact that this is the only keyword that showed discernable peaks in views.

The noted relationship of search volume peaks to news headlines echoes previous findings that studied other neurological diseases, which showed that most of the increase in public awareness came from online published articles that reported on the diagnosis and death of celebrities and political figures (as seen in the cases of Ted Kennedy, Beau Biden, and John McCain who all had glioblastomas) (3). Due to the limited improvement in the overall survival of malignant brain tumors, novel approaches and research breakthroughs have a significant pull in increasing public interest on the matter. This can be maximized to increase research funding for translational medicine for CNS tumors.

This study has limitations similar to other infodemiological studies. First is ensuring that data quality issues, which are inherent in infodemiological studies, are minimized. In terms of completeness, there were no missing data identified from the data sources. However, accuracy of the data was difficult to ascertain. The presented data are mostly based on search term entries, which are highly erratic and central validation of the data acquired has not been fully elucidated. Second, search terms were limited in English. This was ameliorated by using the “topic” option for Google Trends, which generally showed an increase in RSV compared to the “search term” option counterpart of each keyword. However, the exact search algorithm is not provided, thus it is difficult to ascertain if all languages are considered. Thirdly, the bases for search volume were concentrated on Google and Wikipedia, thus there is a possibility of over- or underestimation secondary to missed out data from other region- or cultural-specific search engines. One possible solution to this is using newly developed Internet surveillance systems that could be utilized to detect upcoming epidemics based on search terms like the DiNer-On Building Multilingual Disease-News Profiler (35, 36). Next is the likelihood of increasing search hits by referencing a past event related to brain tumors in online posts about international current events as evidenced in the November 2020 peak corresponding to the election of Joe Biden (father of Beau Biden) as the president of the United States of America. Lastly, it is pertinent to entertain the prospect that analysis of online search volume trends might only be applicable to certain types of medical conditions.

## Conclusion

Online interest on brain tumors have shown stable values using Google and Wikipedia data without significant trends that could imply a general decrease or increase in search queries. Most popular search terms may reflect the incidence of brain tumor subtypes with a predilection to glioblastomas. Peaks in search volume for “brain tumor”, “brain cancer”, and “glioblastoma” can be attributed to publicity garnered from the poor outcomes of famous Western personalities in the past 10 years. These peaks were not directly correlated to actual increase in incidence or prevalence of brain tumors. Therefore, infodemiological data on brain tumors may not be appropriately used to assess worldwide online health-seeking behaviors on central nervous system neoplasms contrary to preliminary studies done on communicable diseases.

## Data Availability Statement

The original contributions presented in the study are included in the article/**Supplementary Material**. Further inquiries can be directed to the corresponding author.

## Author Contributions

MM: Conceptualization, data curation, formal analysis, interpretation of data, writing-original draft, writing-review, and editing. AE: Conceptualization, data curation, formal analysis, interpretation of data, writing-original draft, writing-review, and editing. RJ: Conceptualization, data curation, formal analysis, interpretation of data, writing-original draft, writing-review, and editing. All authors contributed to the article and approved the submitted version.

## Conflict of Interest

The authors declare that the research was conducted in the absence of any commercial or financial relationships that could be construed as a potential conflict of interest.

## Publisher’s Note

All claims expressed in this article are solely those of the authors and do not necessarily represent those of their affiliated organizations, or those of the publisher, the editors and the reviewers. Any product that may be evaluated in this article, or claim that may be made by its manufacturer, is not guaranteed or endorsed by the publisher.
