# Supplementary material for: Brain Tumor Infodemiology: Worldwide Online Health-Seeking Behavior Using Google Trends and Wikipedia Pageviews
Source: Front Oncol. 2022 Apr 19;12:855534. doi: 10.3389/fonc.2022.855534 (PMC9061992; doi:10.3389/fonc.2022.855534)
Supplement: Supplementary file 1 [file Table_1.docx]

**Supplementary Table 1**. Additional parameters from Google Trends per keyword.

| **Google search term** | **Interest by region**  **(Top 10)** | **RSV** | **Related queries**  **(Top 10)** | **RSV** |
| --- | --- | --- | --- | --- |
| “Brain tumor” | Jamaica  United States  Philippines  Nepal  Canada  Pakistan  Australia  United Kingdom  Ghana  South Africa | 100  94  94  90  72  72  65  63  63  62 | Symptoms  Brain tumor symptoms  Tumor symptoms  Tumor in brain  Symptoms of brain tumor  Brain tumor cancer  Cancer  Brain cancer  Brain tumor signs  Tumor in brain symptoms | 100  97  96  62  44  30  29  28  24  21 |
| “Brain tumor” (Disease) | Australia  United Kingdom  Iran  United States  Canada  Indonesia  Ireland  Philippines  Sweden  New Zealand | 100  93  93  89  85  85  84  76  76  71 | Brain  Tumor  Brain tumor  Cancer  Brain cancer  Symptoms brain tumor  Brain tumour  Tumor in brain  Brain tumors  Tumor cerebral | 100  74  55  33  29  15  11  9  9  7 |
| “Brain cancer” | Australia  United States  Canada  Philippines  South Africa  Ireland  United Kingdom  New Zealand  Lebanon  Singapore | 100  76  68  56  54  51  47  46  45  39 | Cancer symptoms  Brain cancer symptoms  Symptoms  Cancer in brain  The brain  Brain tumor  Brain tumor cancer  Cancer of the brain  Symptoms of brain cancer  Symptoms of cancer | 100  99  97  96  90  77  77  49  45  45 |
| “Nervous System Tumor” | United States  India | 100  27 | Brain tumor  Central nervous system  Brain tumor symptoms  Peripheral nervous system | 100  61  25  15 |
| “Glioma” | Nepal  South Korea  United States  Italy  Australia  Puerto Rico  Singapore  Canada  Portugal  United Kingdom | 100  81  79  72  71  66  62  57  56  55 | Brain glioma  Glioma tumor  Glioma cancer  Optic glioma  Pontine glioma  Brain tumor  Low grade glioma  Glioma brain tumor  Glioblastoma  Glioma glioblastoma | 100  75  51  41  40  40  40  39  37  37 |
| “Glioma” (Topic) | Poland  China  Sweden  South Korea  Japan  Italy  Switzerland  Netherlands  United States  Greece | 100  38  21  21  19  19  18  18  17  16 | Glioma  Glejak  Gliomas  Glioma tumor  Gliome  Gliom  **グリオーマ**  Glioma cancer  Glioblastoma  Brain tumor | 100  22  12  9  9  8  6  6  6  6 |
| “Glioblastoma” | United States  Italy  Canada  Puerto Rico  Australia  Ireland  New Zealand  Portugal  South Korea  United Kingdom | 100  97  70  60  58  56  53  53  48  48 | Mutltiforme glioblastoma  Glioblastoma cancer  Cancer  Tumor glioblastoma  Glioblastoma survival  Glioblastoma brain tumor  Brain tumor  Glioblastoma brain cancer  Brain cancer  Glioblastoma treatment | 100  42  41  29  17  17  17  17  17  16 |
| “Glioblastoma” (Genetic Disorder) | Zambia  United States  Germany  Switzerland  Austria  Italy  Canada  Costa Rica  Australia  South Korea | 100  9  8  8  8  8  7  7  7  7 | Glioblastoma  GBM  Multiforme glioblastoma  Glioblastom  Cancer  Glioblastoma cancer  Glioblastome  Glioblastoma tumor  **глиобластома**  Brain tumor | 100  44  19  15  10  7  7  5  4  4 |
| “Glioblastoma Multiforme” | Puerto Rico  Australia  United States  Ireland  Portugal  New Zealand  Spain  Dominican Republic  Canada  Paraguay | 100  82  81  78  77  74  73  66  60  55 | Glioblastoma multiforme cancer  Glioblastoma multiforme brain tumor  Brain tumor  Glioblastoma multiforme treatment  Glioblastoma multiforme grade 4  Glioblastoma grade 4  Glioblastoma multiforme survival  IV glioblastoma multiforme  GBM  Brain cancer | 100  66  61  53  51  48  47  45  41  39 |
| “Astrocytoma” | Nepal  United States  Australia  New Zealand  Philippines  Jordan  Ireland  Canada  Pakistan  United Kingdom | 100  86  76  69  67  58  57  54  51  47 | Anaplastic astrocytoma  Anaplastic  Pilocytic  Astrocytoma tumor  Brain tumor astrocytoma  Brain tumor  Astrocytoma grade 3  Astrocytoma prognosis  Glioblastoma astrocytoma  Glioblastoma | 100  100  98  59  38  36  30  29  28 |
| “Astrocytoma” (Topic) | Germany  Austria  Italy  Switzerland  Ecuador  United States  Mexico  Sweden  Israel  Russia | 100  98  86  84  79  79  77  75  70  68 | Astrocytoma  Astrocitoma  Astrozytom  Tumor  Astrocytoma tumor  **астроцитома**  Astrocytome  Brain tumor  Glioblastoma  Brain tumor astrocytoma | 100  36  15  14  9  8  7  7  7  7 |
| “Oligodendroglioma” | Ireland  United States  Australia  Pakistan  Canada  Portugal  Italy  United Kingdom  South Korea  India | 100  95  94  85  80  77  71  63  55  54 | Anaplastic  Anaplastic oligodendroglioma  Oligodendroglioma tumor  Brain tumor  Astrocytoma  Grade 2 oligodendroglioma  Oligodendroglioma grade 3  Glioblastoma  Glioma  Oligodendroglioma prognosis | 100  95  80  46  39  36  31  31  30  30 |
| “Oligodendroglioma” (Topic) | New Zealand  Ireland  Switzerland  United States  Canada  Australia  South Korea  Italy  Portugal  Pakistan | 100  75  60  59  56  54  51  50  50  44 | Oligodendroglioma  Oligodendrogliom  Anaplastic  Anaplastic oligodendroglioma  Oligodendrogliome  Brain tumor  Astrocytoma  Oligodendroglioma grade 3  Oligodendroglioma grade 2  Oligodendrogliomas | 100  14  14  13  8  6  6  5  4  4 |
| “CNS Lymphoma” | United States  Singapore  Thailand  Canada  India  Australia  United Kingdom | 100  94  67  63  60  60  55 | Primary CNS lymphoma  Brain lymphoma  CNS lymphoma treatment  CNS lymphoma ICD 10  Methotrexate  CNS lymphoma MRI  CNS lymphoma symptoms  CNS lymphoma prognosis  Secondary CNS lymphoma  CNS lymphoma radiology | 100  35  25  24  20  16  16  16  16  14 |
| “Medulloblastoma” | Italy  United States  Jordan  Puerto Rico  Hong Kong  Australia  Canada  Singapore  Philippines  Pakistan | 100  87  87  87  87  75  75  75  75  75 | Medulloblastoma cancer  Medulloblastoma tumor  Medulloblastoma children  Medulloblastoma treatment  Medulloblastoma prognosis  Medulloblastoma survival  Brain tumor  Medulloblastoma brain tumor  Medulloblastoma in children  Ependymoma | 100  87  56  50  50  45  45  43  42  40 |
| “Medulloblastoma” (Topic) | Puerto Rico  Jordan  United States  Italy  Sweden  Switzerland  Germany  Peru  Austria  Australia | 100  70  70  70  70  70  60  60  60  60 | Medulloblastoma  Meduloblastoma  Medulloblastom  Medulloblastoma tumor  Medulloblastoma cancer  **髄 芽 腫**  **médulloblastome**  medullablastoma  Medulloblastome | 100  22  12  6  5  4  3  4  3  3 |
| “Meningioma” | Italy  Nepal  Puerto Rico  United States  Portugal  Australia  Philippines  New Zealand  South Korea  Kenya | 100  83  67  64  62  62  59  54  54  51 | Brain meningioma  Meningioma tumor  Tumor  Brain tumor meningioma  Brain tumor  Meningioma surgery  Meningioma ICD 10  Meningioma cerebral  Meningioma symptoms  Frontal meningioma | 100  99  93  49  47  38  34  33  33  28 |
| “Meningioma” (Medical Condition) | Poland  Italy  France  Israel  Sweden  Switzerland  Greece  Austria  South Korea  Finland | 100  96  82  75  67  67  67  67  64  64 | Meningioma  Meningeom  Brain  Meningiome  Brain meningioma  Tumor meningioma  **髄 膜 腫**  **Oponiak**  **Méningiome** | 100  9  9  9  8  7  6  6  5 |
| “Temozolomide” | South Korea  Ireland  Taiwan  Singapore  New Zealand  India  United States  United Kingdom  Canada  Australia | 100  93  92  91  85  80  74  73  72  70 | Glioblastoma  Temozolomide therapy  Temozolomide side effects  Temodar  Temozolomide price  Temozolomide dose  Temodal  MGMT  Glioma  Temozolomide mechanism | 100  42  42  34  30  30  29  28  27  22 |
| “Temozolomide” (Medication) | Lebanon  Switzerland  Israel  Iran  United States  Bulgaria  Germany  Austria  Romania  Italy | 100  79  77  72  69  63  61  58  57  56 | Temozolomide  Temodal  Temodar  Temozolomida  Glioblastoma  Temozolomid  **темодал**  Temozolamide  Chemotherapy  Temodar side effects | 100  87  64  14  13  11  10  9  6  5 |
| RSV, Relative search volume |  |  |  |  |
